# Bipolar disorder and the risk of cardiometabolic diseases, heart failure, and all-cause mortality: a population-based matched cohort study in South Korea

**DOI:** 10.1038/s41598-024-51757-6

**Published:** 2024-01-22

**Authors:** You-Bin Lee, Hyewon Kim, Jungkuk Lee, Dongwoo Kang, Gyuri Kim, Sang-Man Jin, Jae Hyeon Kim, Hong Jin Jeon, Kyu Yeon Hur

**Affiliations:** 1 Division of Endocrinology and Metabolism, Department of Internal Medicine, Samsung Medical Center, Sungkyunkwan University School of Medicine, 81 Irwon-ro, Gangnam-gu, Seoul, 06351 Republic of Korea; 2 Department of Psychiatry, Depression Center, Samsung Medical Center, Sungkyunkwan University School of Medicine, 81 Irwon-ro, Gangnam-gu, Seoul, 06351 Republic of Korea; 3 Data Science Team, Hanmi Pharm. Co., Ltd., Seoul, Republic of Korea

**Keywords:** Epidemiology, Cardiovascular diseases, Metabolic disorders, Bipolar disorder

## Abstract

The association of bipolar disorder (BD) with the risk of cardiometabolic diseases and premature death in Asians needs to be further determined. Relatively less attention has been paid to heart failure (HF) among cardiometabolic outcomes. We analyzed the Korean National Health Insurance Service database (2002–2018) for this population-based, matched cohort study. The hazards of ischemic stroke, ischemic heart disease (IHD), hospitalization for HF (hHF), composite cardiometabolic diseases, and all-cause mortality during follow-up were compared between individuals with BD (n = 11,329) and 1:1-matched controls without psychiatric disorders among adults without cardiometabolic disease before or within 3 months of baseline. Hazards of outcomes were higher in individuals with BD than in matched controls (adjusted hazard ratios [95% confidence intervals]: 1.971 [1.414–2.746] for ischemic stroke, 1.553 [1.401–1.721] for IHD, 2.526 [1.788–3.567] for hHF, 1.939 [1.860–2.022] for composite cardiometabolic diseases, and 2.175 [1.875–2.523] for all-cause mortality) during follow-up. Associations between BD and outcome hazards were more prominent in younger individuals (p for interaction < 0.02, except for ischemic stroke) and women (p for interaction < 0.04, except for hHF). Screening and preventive measures for cardiometabolic deterioration and early mortality may need to be intensified in individuals with BD, even in young adults, especially women.

## Introduction

Bipolar disorder (BD) is a chronic and recurrent psychiatric disorder and one of the leading causes of disability in younger populations^1,2^. Cognitive and functional impairments can accompany this disorder^1^. Furthermore, previous studies, including meta-analyses, found that people with BD have increased cardiovascular morbidity and mortality, higher all-cause mortality, and shorter life expectancy than the general population^1,3–8^. However, most of these studies were conducted in Western populations in Europe and North America, and the evidence has been limited among Asian populations regarding the relationship between BD and the risk of cardiometabolic outcomes and early mortality. Although population-based studies in Taiwan have reported a higher prevalence and incidence of ischemic heart disease (IHD) and an increased risk of sudden cardiac death (SCD) in individuals with BD than in the general population, the outcomes in these studies were limited to IHD^9^ and SCD^10^. Therefore, whether BD is associated with various cardiometabolic outcomes and early mortality rates in Asian populations remains to be determined.

Among the various cardiometabolic outcomes, as a significant public health issue, heart failure (HF) stands as the leading cause of hospitalization among individuals over the age of 65^11^. Patients with this condition often endure a substantial load of incapacitating symptoms that restrict their daily activities^12–14^. Given the challenges in reversing and curing HF, identifying those at heightened risk of HF development are crucial for formulating successful preventive approaches^13^. In particular in people with BD, HF was revealed to be a major cause of excessive SCD in those aged > 50 years in a recent cohort study^10^. Nevertheless, in terms of the relationship between BD and cardiometabolic outcomes, relatively less attention has been paid to HF among various cardiovascular and metabolic diseases. In a retrospective cohort study that included patients with HF with or without severe mental illness (SMI), including schizophrenia, BD, and severe depression in the United States, SMI was associated with increased mortality among male patients^15^. However, data on the risk of incident HF based on the presence of BD in the absence of premorbid cardiometabolic disease are lacking. In a cross-sectional study involving individuals aged > 45 years, echocardiographic findings were compared between 48 adults with BD and 31 mentally healthy individuals after midlife. People with BD showed more unfavorable cardiac structural measures, suggesting diastolic and systolic dysfunction^16^. However, their study was limited by its small sample size and cross-sectional design, and an association with an incidence of clinically significant HF (e.g. severe cases requiring hospitalization) could not be determined. To address this gap, it is necessary to move beyond the limitations observed in the previous cross-sectional study, which compared echocardiographic structural differences only, and to focus on the incidence of clinically significant HF in a more robust manner through a large-scale longitudinal study.

In this context, our study hypothesized that the presence of BD in Asian populations would significantly alter the risk profiles for various cardiovascular and metabolic diseases, including hospitalization for HF (hHF) and early all-cause mortality. To test this hypothesis, we compared the risks of ischemic stroke, IHD, hHF, composite cardiometabolic diseases, and all-cause mortality during follow-up between individuals with BD and age-and sex-matched controls without psychiatric disorders through a nationwide population-based cohort study in South Korea using the Korean National Health Insurance Service (KNHIS) database. Considering the characteristics of the KNHIS data (unavailability of echocardiographic findings and biomarkers including N-terminal pro-brain natriuretic peptide), we specifically focused on hHF, rather than the simple recording of HF diagnosis as one of the main outcomes. This approach intended not only to reduce the potential for misclassification or over-diagnosis, which may occur with definitions based solely on diagnostic codes, but also to minimize heterogeneity in the severity of disease outcomes and unify the disease spectrum, focusing on clinically significant severe HF cases requiring hospitalization.

## Methods

### Data sources

In this population-based matched cohort study, data from the KNHIS database from January 2002 to December 2018 were analyzed. The KNHIS run by the South Korean government covers all Korean residents as a single mandatory nationwide insurer^17–19^. It was set up as a single-payer system by integrating 375 insurance associations in 2000, and consists of two major health care programs for universal coverage of entire residents in Korea: National Health Insurance covering approximately 97% of the population, and Medical Aid (MA) covering the remaining 3% of the population^18^. Data from MA beneficiaries has been consolidated into a unified NHIS database since 2006^19^. The KNHIS database is an anonymized public database established by KNHIS, which contains data on demographics, household income status, disability, date of death of the deceased, and all types of information on healthcare utilization, including prescriptions, medical or surgical procedures, primary and secondary diagnoses categorized by the International Classification of Diseases-10th Revision (ICD-10), and dates of hospital visits and hospitalization for the entire Korean population including individuals from all ages^18^. These data are interlinked using the de-identified join keys replacing the personal identifiers, and strictly managed by the government to ensure that personal identification is not possible.

The Institutional Review Board (IRB) of Samsung Medical Center approved this study (file number SMC 2019-09-030). This study utilized anonymized public data previously secured for other purposes (the KNHIS claims data) and involved only the review of this data without any additional interventions. Furthermore, the researchers received data from the KNHIS with personal identification information removed, making it impossible to identify the subjects included. Thus, the IRB of Samsung Medical Center granted an informed consent exemption because KNHIS only provided anonymous data to the researchers. After the approval of IRB, we accessed the database under permission of the KNHIS with a limited period of utilization. All methods were performed in accordance with the relevant guidelines and regulations.

### Participant selection and 1:1 exact matching

We used a previously extracted sample population of people with major psychiatric disorders from the KNHIS database. This sample population had been constructed to investigate the association between various major psychiatric disorders and physical illnesses. From all individuals first diagnosed with major psychiatric disorders (BD, depression, or psychotic disorders) between 2003 and 2017, a representative sample cohort comprising 20% of the total sample was randomly selected with systematic stratified random sampling with proportional allocation within each stratum. The strata were constructed according to age, sex, residential area, and level of household income. In this sample population, 27,035 individuals who had first been diagnosed with BD between 2003 and 2017 were selected (Supplementary Fig. 1). BD was defined as the presence of the following: (1) recordings of F30–31 as primary diagnoses and (2) one or more claims for the prescription of psychiatric medications (mood stabilizers, antidepressants, and/or antipsychotics). Those with at least one recording of ICD-10 codes F20–29 (psychotic disorder codes) were excluded. The incidence date of BD was defined as the date on which psychiatric medications (mood stabilizers, antidepressants, and/or antipsychotics) were prescribed for the first time under diagnostic codes for BD (F30–31) or depression (F32–33) in individuals who meet the definition of BD. For comparison, we constructed a representative 20% sample cohort of healthy controls who had never been diagnosed with major psychiatric disorders (BD, depression, or psychotic disorder) between 2002 and 2018 (n = 405,111) after systematically stratified random sampling according to age, sex, residential region, and household income level. The index date (baseline) was set as the incidence date of BD for individuals with BD and January 1, 2003, for healthy controls without major psychiatric disorders. Among the sample population with BD and individuals without major psychiatric disorders, we excluded those who died within 90 days after baseline, those who had before or within 3 months after baseline, claims under codes for cardiometabolic diseases or procedures for coronary diseases, those who had been admitted for transient ischemic attack (G45) before or within 3 months after baseline, and those aged < 18 years at baseline (Supplementary Fig. 1). Through this process, we selected 320,482 adults without metabolic or cardiovascular diseases (CVDs). Of these, 11,350 were individuals with BD and the remaining 309,132 were healthy controls who had never been diagnosed with major psychiatric disorders. Based on age and sex, an exact 1:1 matching was applied to participants with BD and controls without major psychiatric disorders. Finally, 11,329 individuals with BD and 11,329 matched controls without major psychiatric disorders were selected for analysis.

### Outcomes and follow-up

The outcomes of interest were incident ischemic stroke, IHD, hHF, a composite of all cardiometabolic diseases, and all-cause mortality during follow-up. Ischemic stroke was determined by recording ICD-10 code I63 at admission with claims for brain computed tomography or magnetic resonance imaging^20,21^. Definitions of IHD and hHF are presented in Supplementary Table 1. Cardiometabolic diseases include type 2 diabetes, obesity, dyslipidemia, hypertension, IHD, cerebrovascular disease, atherosclerosis, aortic aneurysm and dissection, arterial embolism and thrombosis, and hHF. Definitions of individual components of cardiometabolic diseases are summarized in Supplementary Table 1. All-cause mortality during follow-up indicated death from any cause, including suicide or accident, as well as physical disease. The study population was followed up from baseline until the date of death, outcome development, or December 31, 2018, whichever occurred first.

### Measurements and definitions

The Charlson Comorbidity Index (CCI) was determined using established methods^22^ based on the diagnostic codes presented in a previous report^23^. The presence of disability at baseline was determined using anonymous records from the National Disability Registry System. In South Korea, people with mental, intellectual, or physical disabilities diagnosed by relevant medical specialists are registered on the registry system according to the Korean Disability Act^24^. Among individuals with BD, a history of admission to the psychiatry department and use of psychiatric medications (mood stabilizers, antidepressants, antipsychotics, benzodiazepines, stimulants, and zolpidem) from baseline to the end of follow-up were also examined.

### Statistical analysis

All statistical analyses were performed with SAS Enterprise Guide (version 7.1; SAS Institute, Cary, NC, USA). We considered a two-sided *p*-value < 0.05 as statistically significant. Continuous variables are expressed as mean ± standard deviation and categorical variables as numbers (percentages). The incidence rate for each outcome was calculated as the number of incident cases divided by the total duration of follow-up (person-years). Using Kaplan–Meier curves, cumulative incidence rates of outcomes were compared between individuals with BD and age-and sex-matched controls without major psychiatric disorders; the difference between groups was examined using a log-rank test. Multivariable Cox regression analyses were performed to calculate hazard ratios (HRs) and 95% confidence intervals (CIs) for the outcomes in individuals with BD setting age-and sex-matched controls as the reference. Model 1 was unadjusted, Model 2 was adjusted for CCI, and Model 3 was adjusted for CCI, household income, and disability.

The HRs (95% CIs) for the outcome incidence were compared between individuals with BD and controls without major psychiatric disorders (reference) in the subgroups. The subgroups were constructed according to age (18–39 years, 40–64 years, or ≥ 65 years), sex, quartiles of household income (lowest quartile [Q1] versus Q2–Q4), CCI (< 1 versus ≥ 1), and presence of disability. The *p*-value for the interaction was calculated after examining the potential effect modification by the factors determining the subgroups.

### Sensitivity analyses

We plotted a cumulative incidence function for ischemic stroke, IHD, hHF, and a composite of all cardiometabolic diseases to account for the competing risk by all-cause mortality and estimated sub-distribution HRs using the Fine and Gray method^25^ for the four outcomes after adjusting for the same potential confounders included in the main analyses. Furthermore, we divided individuals with BD based on the history of mood stabilizer treatment during the entire study period and estimated the HRs (95% CIs) for outcome incidence in these subpopulations with BD (individuals never treated with mood stabilizers and those who were treated with mood stabilizers) compared with controls without major psychiatric disorders. We also categorized participants with BD into two subpopulations: (1) those who had been diagnosed with BD at the onset and (2) those who had been initially diagnosed with depression, but whose final diagnoses were later changed to BD. We calculated the hazards of outcomes in these subpopulations of participants with BD compared to controls without major psychiatric disorders. During these sensitivity analyses in subpopulations with BD, the models were adjusted for age and sex and the potential confounders used in the main analyses.

## Results

### Baseline characteristics of the study population

In total, 11,329 individuals with BD and 11,329 matched controls without major psychiatric disorders were included after exact 1:1-matching according to age and sex (Supplementary Fig. 1). The baseline characteristics of the two groups are summarized in Table 1. In both groups, the mean age was 34.06 ± 12.87 years and 38.82% of the individuals were men. Subjects with BD showed higher CCI scores and a higher proportion of individuals with disabilities. Among patients with BD, the uses of mood stabilizers, antidepressants, antipsychotics, benzodiazepines, stimulants, and zolpidem throughout the follow-up period are summarized in Table 1. From baseline to the end of the follow-up period, at least one hospitalization in the psychiatric department was identified in 2.67% of participants with BD.

**Table 1:** Baseline characteristics of the study population.

	Individuals with bipolar disorder	Matched* controls without major psychiatric disorders	*p* value
*n*	11,329	11,329	
Male	4398 (38.82)	4398 (38.82)	1.0000
Age (years)	34.06 ± 12.87	34.06 ± 12.87	1.0000
Age group			
18–39 years	8038 (70.95)	8038 (70.95)	1.0000
40–64 years	2967 (26.19)	2967 (26.19)	
≥ 65 years	324 (2.86)	324 (2.86)	
Household income quartile			
Q1	3034 (26.78)	2742 (24.20)	< 0.0001
Q2	2440 (21.54)	2591 (22.87)	
Q3	2601 (22.96)	2995 (26.44)	
Q4	3254 (28.72)	3001 (26.49)	
CCI	0.28 ± 0.62	0.02 ± 0.18	< 0.0001
CCI categories			
0	8979 (79.26)	11,110 (98.07)	< 0.0001
1	1856 (16.38)	188 (1.66)	
2	394 (3.48)	23 (0.20)	
≥ 3	100 (0.88)	8 (0.07)	
Disability	537 (4.74)	165 (1.46)	< 0.0001
Psychotropic medication uses throughout the follow-up period		N/A	
Mood stabilizer	8489 (74.93)		
Antidepressant	8514 (75.15)		
Antipsychotics	8051 (71.07)		
Benzodiazepine	10,099 (89.14)		
Stimulant	1098 (9.69)		
Zolpidem	5145 (45.41)		
Admission to psychiatry	302 (2.67)		

Values are presented as number (%) or mean ± standard deviation.

*CCI* charlson comorbidity index.

*Exact 1:1 matching based on age and sex.

### Primary analyses

**Figure 1:**
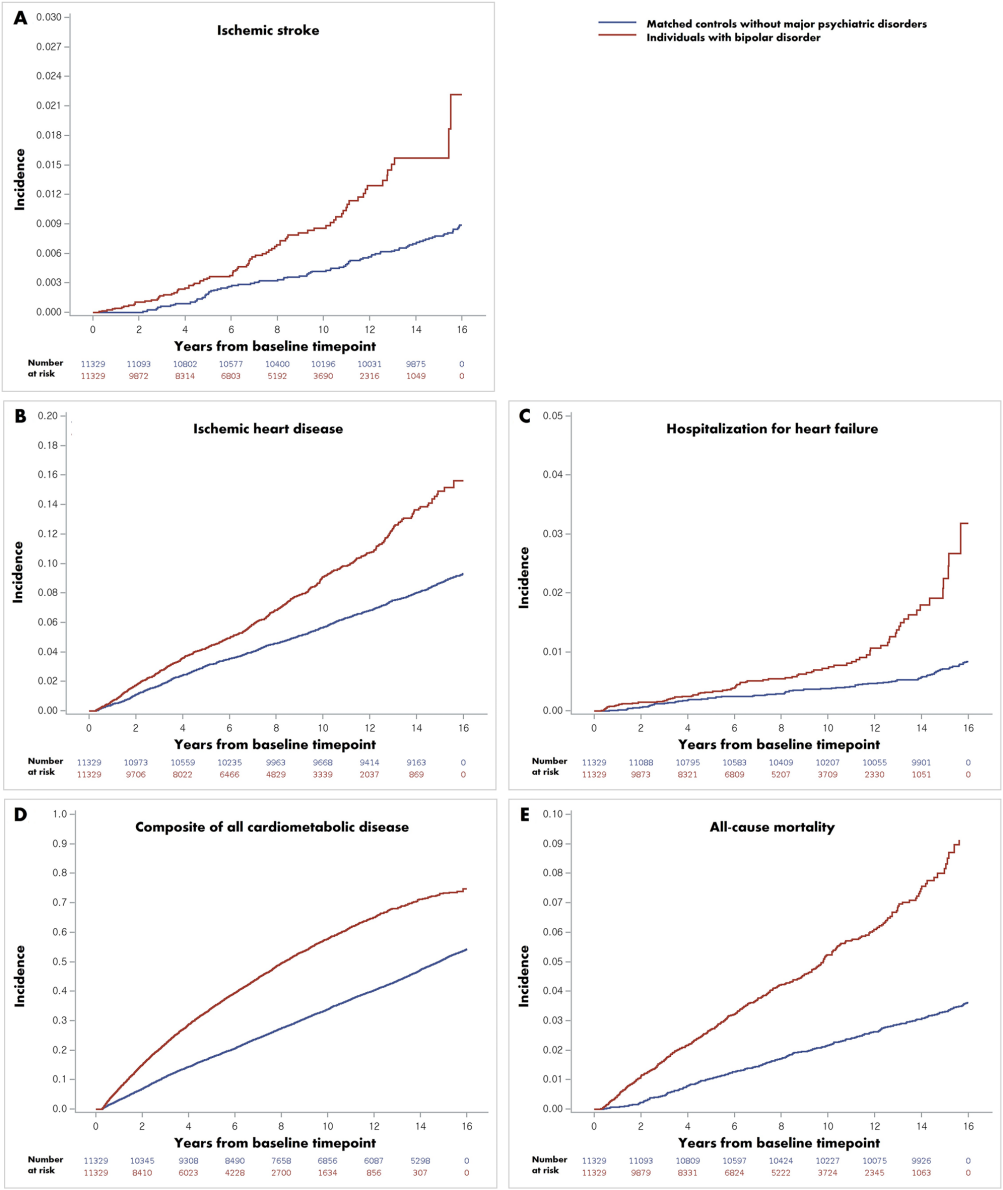
Kaplan–Meier plots of the cumulative incidence of (**A**) ischemic stroke, (**B**) ischemic heart disease, (**C**) hospitalization for heart failure, (**D**) composite of all cardiometabolic diseases, and (**E**) all-cause mortality during follow-up in patients with bipolar disorder *versus* matched controls without major psychiatric disorders.

After a mean of 11.19 years (253,605 person-years), 173 cases of ischemic stroke occurred (Table 2). After a mean of 10.72 years (242,808 person-years), 1757 cases of IHD developed and 169 cases of hHF were detected during a mean of 11.21 years (253,884 person-years). After a mean of 8.11 years (183,835 person-years), 10,832 composite cases of all cardiometabolic diseases were diagnosed. A total of 857 deaths occurred during a mean of 11.23 years (254,349 person-years). The cumulative incidence of all outcomes was higher in participants with BD than in age-and sex-matched controls without major psychiatric disorders (Fig. 1). The hazards of all outcomes were higher in individuals with BD than in matched controls without major psychiatric disorders (adjusted HR [95% CI]: 1.971 [1.414–2.746] for ischemic stroke, 1.553 [1.401–1.721] for IHD, 2.526 [1.788–3.567] for hHF, 1.939 [1.860–2.022] for a composite of all cardiometabolic diseases, and 2.175 [1.875–2.523] for all-cause mortality during follow-up) (Table 2).

**Table 2:** Hazard ratios and 95% confidence intervals for outcome incidences according to presence of bipolar disorder.

Presence of bipolar disorder	Subjects (n)	Events (n)	Follow-up duration (person-years)	Incidence rate (per 1000 person-years)	Hazard ratio (95% confidence interval)		
					Model 1	Model 2	Model 3
Ischemic stroke							
Matched* controls without major psychiatric disorders	11,329	92	167,546	0.55	1 (Ref.)	1 (Ref.)	1 (Ref.)
Individuals with bipolar disorder	11,329	81	86,059	0.94	**2.216 (1.607, 3.057)**	**2.104 (1.515, 2.922)**	**1.971 (1.414, 2.746)**
*p* value					< 0.0001	< 0.0001	< 0.0001
Ischemic heart disease							
Matched* controls without major psychiatric disorders	11,329	981	160,754	6.10	1 (Ref.)	1 (Ref.)	1 (Ref.)
Individuals with bipolar disorder	11,329	776	82,054	9.46	**1.642 (1.486, 1.815)**	**1.600 (1.444, 1.772)**	**1.553 (1.401, 1.721)**
*p* value					< 0.0001	< 0.0001	< 0.0001
Hospitalization for heart failure							
Matched* controls without major psychiatric disorders	11,329	87	167,698	0.52	1 (Ref.)	1 (Ref.)	1 (Ref.)
Individuals with bipolar disorder	11,329	82	86,186	0.95	**2.745 (1.961, 3.843)**	**2.648 (1.880, 3.729)**	**2.526 (1.788, 3.567)**
*p* value					< 0.0001	< 0.0001	< 0.0001
Composite of all cardiometabolic disease							
Matched* controls without major psychiatric disorders	11,329	5769	124,342	46.40	1 (Ref.)	1 (Ref.)	1 (Ref.)
Individuals with bipolar disorder	11,329	5063	59,493	85.10	**2.046 (1.965, 2.131)**	**1.965 (1.885, 2.048)**	**1.939 (1.860, 2.022)**
*p* value					< 0.0001	< 0.0001	< 0.0001
All-cause mortality during follow-up							
Matched* controls without major psychiatric disorders	11,329	385	167,968	2.29	1 (Ref.)	1 (Ref.)	1 (Ref.)
Individuals with bipolar disorder	11,329	472	86,381	5.46	**2.517 (2.180, 2.905)**	**2.352 (2.031, 2.723)**	**2.175 (1.875, 2.523)**
*p* value					< 0.0001	< 0.0001	< 0.0001

Model 1: Unadjusted.

Model 2: Adjusted for CCI.

Model 3: Adjusted for CCI, household income, and disability.

The hazard ratio values that are statistically significant are in bold.

*CCI* charlson comorbidity index

*Exact 1:1 matching based on age and sex.

### Subgroup analyses

**Figure 2:**
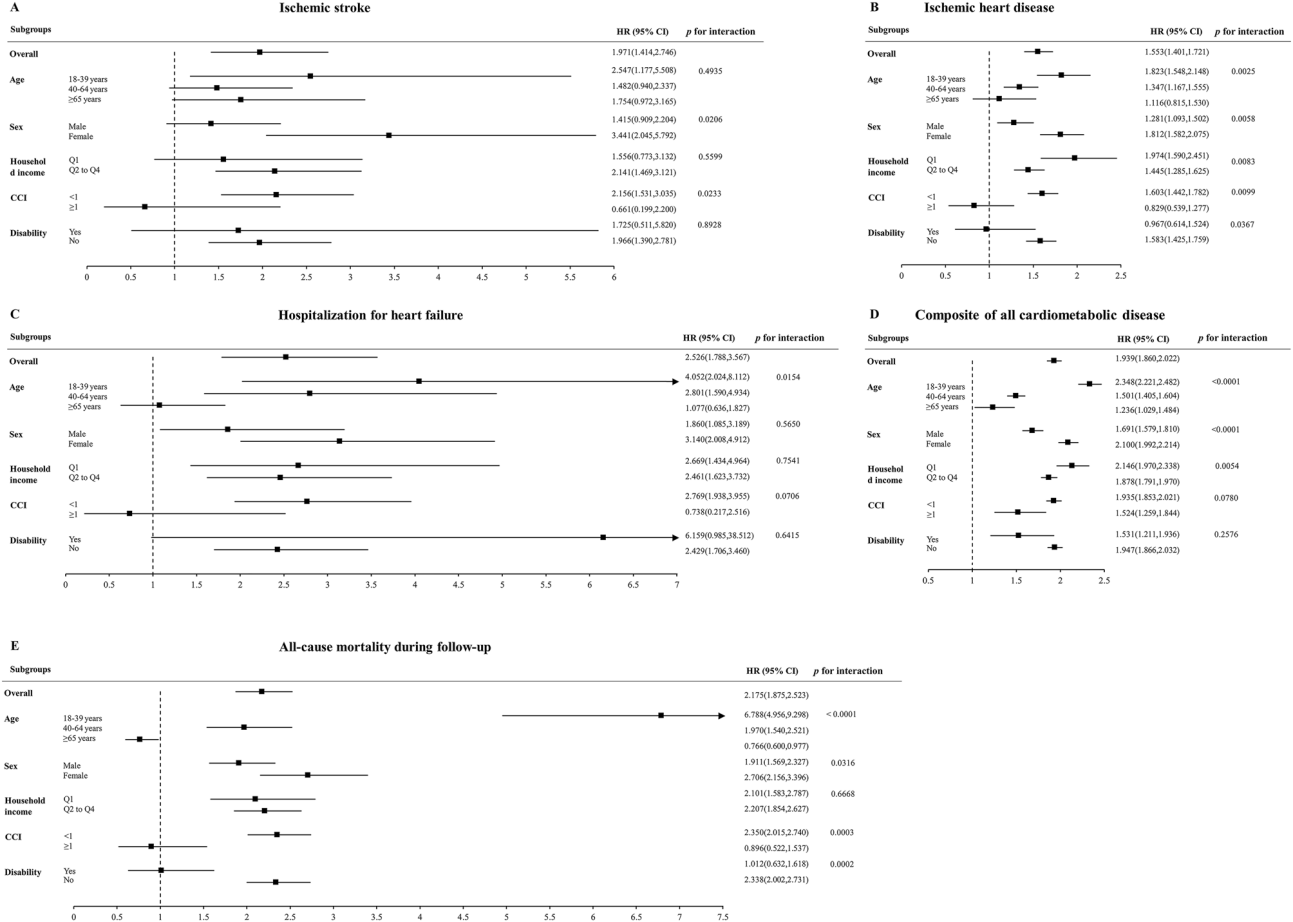
Subgroup analyses for the hazard of (**A**) ischemic stroke, (**B**) ischemic heart disease, (**C**) hospitalization for heart failure, (**D**) composite of all cardiometabolic diseases, and (**E**) all-cause mortality during follow-up in patients with bipolar disorder *versus* matched controls without major psychiatric disorders.

The HRs (95% CIs) of outcome incidence in participants with BD compared to controls without major psychiatric disorders were analyzed in subgroups classified by age, sex, household income quartile, CCI, and presence of disability (Fig. 2). Subjects with BD exhibited a higher hazard of outcome than their matched controls in all subgroups except those aged ≥ 65 years, those with CCI ≥ 1, and disabled people (for outcomes of ischemic stroke, IHD, hHF, and all-cause mortality), as well as those aged 40–64 years, men, those with the lowest quartile of household income (for outcomes of ischemic stroke). The association between BD and the hazards of all outcomes, except ischemic stroke, was more prominent in younger individuals (*p* for interaction < 0.02). The association between BD and the hazards of all outcomes, except hHF, was more pronounced in women (*p* for interaction < 0.04). Regarding the outcomes of ischemic stroke, IHD, and all-cause mortality, the increased hazards in individuals with BD compared to matched controls were more prominent in subjects with CCI < 1 (*p* for interaction < 0.03). Furthermore, the association between BD and hazards of IHD and the composite of all cardiometabolic diseases was more pronounced in individuals in the lowest household income quartile (*p* for interaction < 0.01). Regarding the outcomes of IHD and all-cause mortality during follow-up, the association between BD and outcomes was more prominent in participants without disabilities (*p* for interaction < 0.04).

### Sensitivity analyses

When all-cause mortality was considered competing events, the corresponding cumulative incidence function and sub-distribution HRs for ischemic stroke, IHD, hHF, and the composite of all cardiometabolic diseases were consistent with the findings of the main analyses (Supplementary Fig. 2 and Supplementary Table 2).

Increased hazards of outcomes in individuals with BD compared to controls without major psychiatric disorders were consistently observed regardless of the history of mood stabilizer treatment (Supplementary Table 3). When we categorized individuals with BD into those who had initially been diagnosed with depression and those who had been diagnosed with BD from the onset, both subpopulations consistently showed significantly higher outcome hazards than the controls without major psychiatric disorders (Supplementary Table 4).

## Discussion

Although the study population consisted of only low-risk individuals without baseline hypertension, dyslipidemia, diabetes, or any other cardiometabolic or vascular diseases, in this population-based nationwide cohort study that included 11,329 adults with BD and 1:1 age-and sex-matched controls without psychiatric disorders, BD was associated with an increased risk of ischemic stroke, IHD, hHF, composite cardiometabolic diseases, and all-cause mortality during follow-up. These findings were consistent regardless of whether mood stabilizers had been used or not and whether the initial diagnoses were depression or BD at disease onset in individuals with BD. Regarding the outcomes of ischemic stroke, IHD, hHF, and composite cardiometabolic diseases, sub-distribution HRs considering all-cause mortality as a competing event showed consistent findings. In the subgroup analyses, the association was more prominent in younger individuals and women.

Our findings indicate that BD may be associated with an increased risk of adverse cardiometabolic outcomes and early mortality in Koreans, an Asian population. Although this observational study could not clarify the exact mechanism, as possible hypotheses, several factors as follows may have affected the excess risk of cardiometabolic outcomes and premature all-cause mortality in participants with BD^2^. First, people with SMI, including those with BD, may have unhealthy lifestyles, such as poor diet, lack of exercise, and smoking, which leads to an increasing burden of CVD risk factors^2,26^. Second, psychotropic medications used in individuals with BD, including antipsychotics, mood stabilizers, and antidepressants, may have contributed. Although these pharmacological interventions relieve the overall symptom burden of mental illness and may result in better help-seeking behaviors and lifestyle choices, several psychotropic medications may accompany metabolic adverse effects^2^. Third, in people with BD, lack of knowledge about health-related topics, difficulties in seeking adequate care for physical diseases and following recommendations from healthcare providers, and poor adherence to therapy can be obstacles in recognizing and receiving appropriate care for physical health^2,27^. Fourth, as healthcare system factors, people with SMI, including BD, may often experience social deprivation and unfavorable attitudes when seeking help with physical care, leading to disparities in access to healthcare, lower use of diagnostic procedures, and delayed initiation of therapy for physical diseases, including cardiometabolic diseases^2^, all of which may lead to the increased risk of adverse cardiometabolic outcomes and premature mortality.

Our findings show that BD may also be associated with an increased risk of hHF. Previously, two small cross-sectional studies compared systolic and diastolic functions in individuals with BD and healthy controls using echocardiography^16,28^, which demonstrated inconsistent findings. No significant differences were observed in left ventricular systolic and diastolic functions in a case-control study conducted in Türkiye, which included 30 healthy volunteers and 30 individuals with BD on lithium therapy in the therapeutic range^28^. However, in a more recent study conducted among 48 participants with BD and 31 mentally healthy adults aged > 45 years in Taiwan, compared to controls, participants with BD showed more unfavorable echocardiographic findings, indicating diastolic and systolic dysfunction^16^. Herein, we provide evidence of an increased risk of clinically significant HF according to the presence of BD in the absence of premorbid cardiometabolic diseases in a large-scale, longitudinal, matched cohort study. In addition to the aforementioned patient and healthcare system factors that can mediate excess outcome risks in individuals with BD, there have been reports on inflammatory alterations^29^, dysregulation of mitochondrial function^30^, oxidative stress^30,31^, and autonomic dysregulation^32,33^ in people with BD. These theories may mechanistically elucidate the higher risk of hHF in people with BD observed in our analyses, although the exact mechanism remains unclarified in this observational study.

The excess risk of outcomes associated with BD was more pronounced in younger individuals and women, implying that the effects of BD on the risk of cardiometabolic disease and early all-cause mortality may be more prominent among these subgroups. Similarly, in a nationwide study in Denmark that showed an elevated mortality rate in people with BD (relative to the general population) represented by increased standardized mortality ratios (SMRs), the highest SMR was observed in the youngest population^3^. In people with BD, accelerated aging and consequent chronologically earlier development of risk factors and physical diseases due to an advanced biological age have been suggested^34,35^. Regarding the interaction by age, the older biological age in people with BD due to accelerated aging may have exerted a more prominent influence on younger individuals on the risk of premature mortality and cardiovascular diseases. Furthermore, similar to our results that show a more prominent association in women, in a cross-sectional analysis of 293 participants with BD and 257,380 psychiatrically healthy controls in the UK Biobank, a two-to three-fold stronger association was observed between BD and the rates of coronary artery disease, HF, and essential hypertension in women than in men after adjusting for age^36^. Regarding this gender-specific interaction, we considered following factors as possible hypotheses although exact mechanism cannot be elucidated in this observational study. First, the higher age at cardiovascular disease onset in women compared to men in the general population, along with a greater burden of cardiovascular risk factors at disease onset in women^37^, may have influenced the finding. Due to the protective effects of estrogen, women, compared to men, usually exhibit a lower age-specific risk of cardiovascular disease until menopause^37^, and the impact of BD may have been greater in women, who generally have a lower overall risk. Second, the detrimental effects of unhealthy lifestyles, such as smoking, which are more likely to be prevalent in patients with BD, may have a more pronounced impact in women compared to men. In previous reports, the association between smoking and CVD risk was found to be stronger in women than men^37,38^, probably due to a smoking-induced downregulation of estrogen-dependent vasodilatation of the endothelial wall^37,39^. Third, the burden of female-specific risk factors such as polycystic ovary syndrome (PCOS) and contraceptive pills might have contributed considering the close link between PCOS and BD in women^40^. Lastly, women with BD might have experienced more severe disparities in accessing healthcare utilization, or more discrimination in social factors (e.g. employment^41^ and/or education) by the diagnosis of BD, compared to men with BD.

The strengths of our study include the use of a representative nationwide dataset operated by the South Korean government, where incidences of outcome measures were detected without exception under the single-insurer system in South Korea; 1:1 exact matching based on age and sex; various subgroup analyses; and sensitivity analyses, which yielded consistent findings. This study has some limitations. First, only Korean adults with low cardiovascular risk were included in the current study. Therefore, caution should be exercised before extrapolating our findings to populations of different ethnicities or those with multiple baseline cardiometabolic risk factors such as hypertension, diabetes, and/or dyslipidemia. Second, as an observational study, it is inevitably limited in clarifying causal relationships. However, to ensure a temporal relationship between exposure and outcome incidence and to minimize the potential reverse causality effect, we excluded individuals with any metabolic or cardiovascular disease before or within three months after baseline. Third, using the current KNHIS database, the specific cause of death could not be determined, although cardiovascular and non-cardiovascular mortality, including those from suicide or accidents, may have varied clinical implications. However, a considerable proportion of premature mortality in participants with BD in the current study was expected to be due to natural death from physical conditions, including cardiovascular causes. Although unnatural death rates from suicide or accidents have been reported to be higher in people with BD than in the general population, physical conditions, including CVDs, represent the main cause of death among people with BD^2^, and according to a cohort study in Sweden, approximately one-third of individuals with BD died from CVDs, prevailing the external causes^6^. Fourth, the effect of unmeasured confounders may remain although we applied exact 1:1-matching for age and sex between groups, adjusted for measured potential confounders, and conducted various subgroup analyses. Particularly, due to the absence of relevant information in the KNHIS database or limitations in accessing the database for a specific period, it was not possible to examine the association based on mood symptom burden, lifestyle factors, or generations of antipsychotics.

## Conclusions

In this population-based nationwide real-world cohort study with exact 1:1 matching for age and sex, BD was associated with an increased risk of ischemic stroke, IHD, hHF, composite cardiometabolic diseases, and premature all-cause mortality. This association was particularly pronounced in younger individuals and women. These findings indicate that regular screening for cardiometabolic deterioration and preventive measures for cardiovascular disease and early mortality should be intensified in individuals with BD, even in young adults, especially women. Somatic health care to prevent cardiometabolic deterioration, including patient education, lifestyle modification, and cardioprotective pharmacological interventions incorporated with psychiatric care, may help optimize outcomes in people with BD.

### Supplementary Information

Supplementary Information.

## Data Availability

The data that support the findings of this study are available from the Korean National Health Insurance Service (KNHIS) but restrictions apply to the availability of these data, which were used under license for the current study, and so are not publicly available. Data are however available from the corresponding authors upon reasonable request and with permission of the KNHIS.

## Abbreviations

BD: Bipolar disorder
CABG: Coronary artery bypass graft
CCI: Charlson Comorbidity Index
CI: Confidence interval
CVD: Cardiovascular disease
HF: Heart failure
hHF: Hospitalization for HF
HR: Hazard ratio
ICD-10: International Classification of Diseases-10th Revision
IHD: Ischemic heart disease
KNHIS: Korean National Health Insurance Service
PCI: Percutaneous coronary intervention
PTCA: Percutaneous transluminal coronary angioplasty
SCD: Sudden cardiac death
SMI: Severe mental illness
SMR: Standardized mortality ratio

## References

[1] Vieta E (2018). Bipolar disorders. Nat. Rev. Dis. Prim..

[2] Nielsen RE, Banner J, Jensen SE (2021). Cardiovascular disease in patients with severe mental illness. Nat. Rev. Cardiol..

[3] Staudt Hansen P (2019). Increasing mortality gap for patients diagnosed with bipolar disorder-A nationwide study with 20 years of follow-up. Bipolar Disord..

[4] Hayes JF, Marston L, Walters K, King MB, Osborn DPJ (2017). Mortality gap for people with bipolar disorder and schizophrenia: UK-based cohort study 2000–2014. Br. J. Psychiatry.

[5] Hayes JF, Miles J, Walters K, King M, Osborn DP (2015). A systematic review and meta-analysis of premature mortality in bipolar affective disorder. Acta Psychiatr. Scand..

[6] Westman J (2013). Cardiovascular mortality in bipolar disorder: A population-based cohort study in Sweden. BMJ Open.

[7] Callaghan RC, Khizar A (2010). The incidence of cardiovascular morbidity among patients with bipolar disorder: A population-based longitudinal study in Ontario, Canada. J. Affect. Disord..

[8] Foroughi M (2022). Association of bipolar disorder with major adverse cardiovascular events: A population-based historical cohort study. Psychosom. Med..

[9] Hsu JH, Chien IC, Lin CH (2021). Increased risk of ischemic heart disease in patients with bipolar disorder: A population-based study. J. Affect. Disord..

[10] Chen PH (2020). Incidence and risk factors of sudden cardiac death in bipolar disorder across the lifespan. J. Affect. Disord..

[11] Benjamin EJ (2019). Heart disease and stroke statistics-2019 update: A report from the american heart association. Circulation.

[12] Kosiborod MN (2020). Effects of dapagliflozin on symptoms, function, and quality of life in patients with heart failure and reduced ejection fraction: Results from the DAPA-HF trial. Circulation.

[13] Lee YB (2020). Hospitalization for heart failure incidence according to the transition in metabolic health and obesity status: A nationwide population-based study. Cardiovasc. Diabetol..

[14] Bui AL, Horwich TB, Fonarow GC (2011). Epidemiology and risk profile of heart failure. Nat. Rev. Cardiol..

[15] Polcwiartek C (2021). Clinical heart failure among patients with and without severe mental illness and the association with long-term outcomes. Circ. Heart Fail..

[16] Chen PH (2022). Echocardiographic study of cardiac structure and function in people with bipolar disorder after midlife. J. Affect. Disord..

[17] Kim HK, Song SO, Noh J, Jeong IK, Lee BW (2020). Data configuration and publication trends for the korean national health insurance and health insurance review & assessment database. Diabetes Metab. J..

[18] Cheol Seong S (2017). Data resource profile: The national health information database of the national health insurance service in South Korea. Int. J. Epidemiol..

[19] Lee YH, Han K, Ko SH, Ko KS, Lee KU (2016). Data analytic process of a nationwide population-based study using national health information database established by national health insurance service. Diabetes Metab. J..

[20] Kim MK (2017). Cholesterol variability and the risk of mortality, myocardial infarction, and stroke: A nationwide population-based study. Eur. Heart J..

[21] Kim MK (2019). Blood pressure and development of cardiovascular disease in koreans with type 2 diabetes mellitus. Hypertension.

[22] Charlson ME, Pompei P, Ales KL, MacKenzie CR (1987). A new method of classifying prognostic comorbidity in longitudinal studies: Development and validation. J. Chron. Dis..

[23] Sundararajan V (2004). New ICD-10 version of the charlson comorbidity index predicted in-hospital mortality. J. Clin. Epidemiol..

[24] Jung I (2020). The prevalence and risk of type 2 diabetes in adults with disabilities in Korea. Endocrinol. Metab. (Seoul).

[25] Fine JP, Gray RJ (1999). A proportional hazards model for the subdistribution of a competing risk. J. Am. Stat. Assoc..

[26] Mitchell AJ, Vancampfort D, De Hert M, Stubbs B (2015). Do people with mental illness receive adequate smoking cessation advice? A systematic review and meta-analysis. Gen. Hosp. Psychiatry.

[27] De Hert M (2011). Physical illness in patients with severe mental disorders. II. Barriers to care, monitoring and treatment guidelines, plus recommendations at the system and individual level. World Psychiatry.

[28] Zencir C (2015). Evaluation of left ventricular systolic and diastolic functions in bipolar patients during lithium therapy. Int. J. Clin. Exp. Med..

[29] Fries GR, Walss-Bass C, Bauer ME, Teixeira AL (2019). Revisiting inflammation in bipolar disorder. Pharmacol. Biochem. Behav..

[30] Morris G (2017). A model of the mitochondrial basis of bipolar disorder. Neurosci. Biobehav. Rev..

[31] Hatch J (2015). Cardiovascular and psychiatric characteristics associated with oxidative stress markers among adolescents with bipolar disorder. J. Psychosom. Res..

[32] Henry BL, Minassian A, Paulus MP, Geyer MA, Perry W (2010). Heart rate variability in bipolar mania and schizophrenia. J. Psychiatr. Res..

[33] Quintana DS (2016). Reduced heart rate variability in schizophrenia and bipolar disorder compared to healthy controls. Acta Psychiatr. Scand..

[34] Rizzo LB (2014). The theory of bipolar disorder as an illness of accelerated aging: Implications for clinical care and research. Neurosci. Biobehav. Rev..

[35] Yang F (2018). Further evidence of accelerated aging in bipolar disorder: Focus on GDF-15. Transl. Neurosci..

[36] Ortiz A (2022). Sex-specific associations between lifetime diagnosis of bipolar disorder and cardiovascular disease: A cross-sectional analysis of 257,673 participants from the UK biobank. J. Affect. Disord..

[37] Gao Z, Chen Z, Sun A, Deng X (2019). Gender differences in cardiovascular disease. Med. Novel Technol. Dev..

[38] Grundtvig M, Hagen TP, German M, Reikvam A (2009). Sex-based differences in premature first myocardial infarction caused by smoking: Twice as many years lost by women as by men. Eur. J. Cardiovasc. Prev. Rehabil..

[39] Vanhoutte PM, Shimokawa H, Tang EH, Feletou M (2009). Endothelial dysfunction and vascular disease. Acta Physiol. (Oxf).

[40] Jiang B, Kenna HA, Rasgon NL (2009). Genetic overlap between polycystic ovary syndrome and bipolar disorder: The endophenotype hypothesis. Med. Hypotheses.

[41] Holm M, Taipale H, Tanskanen A, Tiihonen J, Mitterdorfer-Rutz E (2021). Employment among people with schizophrenia or bipolar disorder: A population-based study using nationwide registers. Acta Psychiatr. Scand..

