# Supplementary material for: Bipolar disorder and the risk of cardiometabolic diseases, heart failure, and all-cause mortality: a population-based matched cohort study in South Korea
Source: Sci Rep. 2024 Jan 22;14:1932. doi: 10.1038/s41598-024-51757-6 (PMC10803345; doi:10.1038/s41598-024-51757-6)
Supplement: Supplementary file 1 — Supplementary Information. [file 41598_2024_51757_MOESM1_ESM.docx]

**Supplementary Figure Legends**

**Supplementary Fig. 1.** Flow diagram of participant selection.


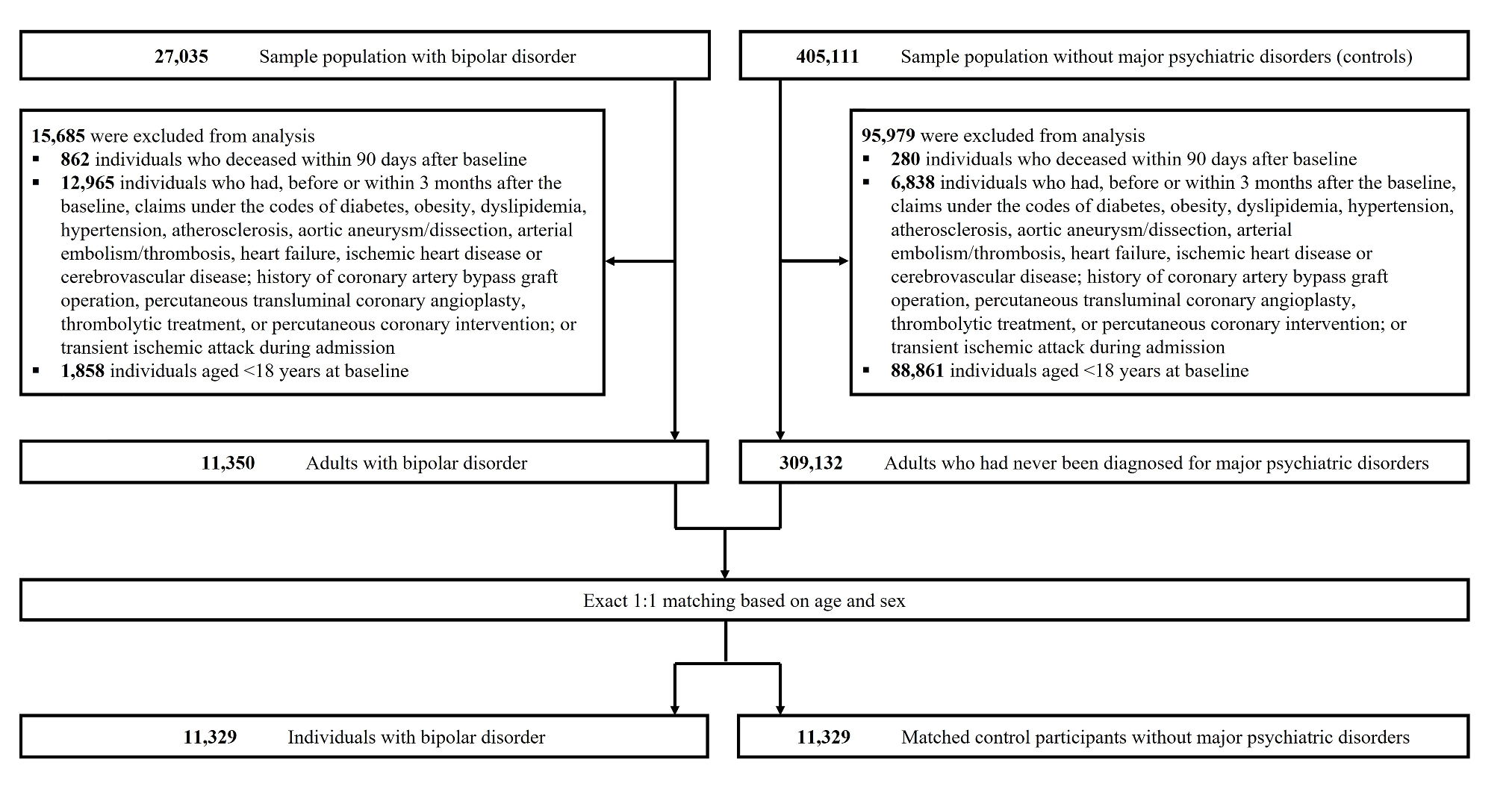


**Supplementary Fig. 2.** Cumulative incidence function of **(A)** ischemic stroke, (**B**) ischemic heart disease, (**C**) hospitalization for heart failure, and (**D**) the composite of all cardiometabolic diseases according to the presence of bipolar disorder, accounting for all-cause mortality as a competing event.


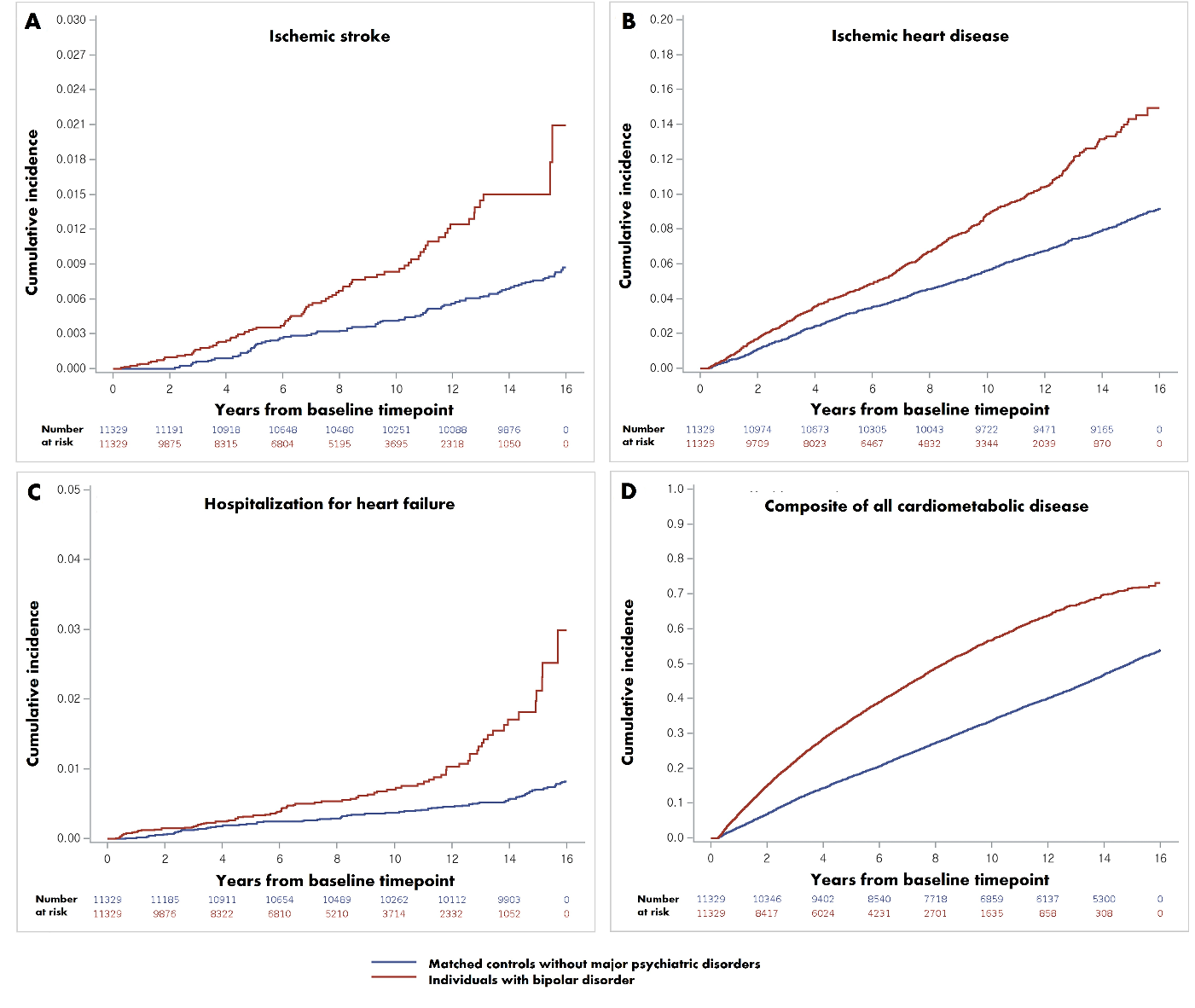


**Supplementary Table 1.** Definition of each component of cardiometabolic diseases

| **Components of cardiometabolic diseases** | **Definition** |
| --- | --- |
| Type 2 diabetes^1-3^ | One or more claims per year for the prescription of anti-diabetes medications with ICD-10 codes E11–14 |
| Obesity | Recorded ICD-10 code E66 |
| Dyslipidemia | Recorded ICD-10 code E78 |
| Hypertension | Recorded ICD-10 codes I10–I15 |
| Ischemic heart disease | Recorded ICD-10 codes I20–25 or claims for percutaneous transluminal coronary angioplasty, percutaneous coronary intervention, thrombolytic treatment, or coronary artery bypass graft |
| Cerebrovascular disease | Claims for G45 during hospitalization or recorded ICD-10 codes I61–69 |
| Atherosclerosis | Recorded I70 |
| Aortic aneurysm and dissection | Recorded I71–72 |
| Arterial embolism and thrombosis | Recorded I74 |
| Hospitalization for heart failure^3-5^ | The first hospitalization with a primary diagnosis of ICD-10 code I50 |

**References**

1. Noh, J., Han, K. D., Ko, S. H., Ko, K. S. & Park, C. Y. Trends in the pervasiveness of type 2 diabetes, impaired fasting glucose and co-morbidities during an 8-year-follow-up of nationwide Korean population. *Sci. Rep*. **7**, 46656. http://doi.org/10.1038/srep46656 (2017).

2. Lee, Y. B. *et al*. Risk of type 2 diabetes according to the cumulative exposure to metabolic syndrome or obesity: A nationwide population-based study. *J. Diabetes Investig*. **11,** 1583-1593. http://doi.org/10.1111/jdi.13304 (2020).

3. Lee, Y. B. *et al*. Risk of early mortality and cardiovascular disease in type 1 diabetes: a comparison with type 2 diabetes, a nationwide study. *Cardiovasc. Diabetol*. **18,** 157. http://doi.org/10.1186/s12933-019-0953-7 (2019).

4. Lee, Y. B. *et al*. Hospitalization for heart failure incidence according to the transition in metabolic health and obesity status: a nationwide population-based study. *Cardiovasc. Diabetol*. **19,** 77. http://doi.org/10.1186/s12933-020-01051-2 (2020).

5. Lee, Y. B. *et al*. Severe hypoglycemia and risk of hospitalization for heart failure in adults with diabetes treated with oral medications with or without insulin: A population-based study. *Diabetes Res Clin Pract*. **192,** 110083. http://doi.org/10.1016/j.diabres.2022.110083 (2022).

**Supplementary Table 2.** Sub-distribution hazard ratios and 95% confidence intervals for cardiometabolic diseases according to the presence of bipolar disorder, accounting for all-cause mortality as a competing event

| **Presence of bipolar disorder** | **Subjects (n)** | **Events (n)** | **Follow-up duration (person-years)** | **Incidence rate (per 1,000 person-years)** | **Hazard ratio (95% confidence interval)** | | |
| --- | --- | --- | --- | --- | --- | --- | --- |
|  |  |  |  |  | **Model 1** | **Model 2** | **Model 3** |
| **Ischemic stroke** | | | | | | | |
| Matched^*^ controls without major psychiatric disorders | 11329 | 92 | 167546 | 0.55 | 1 (Ref.) | 1 (Ref.) | 1 (Ref.) |
| Individuals with bipolar disorder | 11329 | 81 | 86059 | 0.94 | **2.074 (1.509,2.850)** | **1.985 (1.425,2.767)** | **1.858 (1.328,2.600)** |
| *P* value | | | | | <0.0001 | <0.0001 | 0.0003 |
| **Ischemic heart disease** | | | | | | | |
| Matched^*^ controls without major psychiatric disorders | 11329 | 981 | 160754 | 6.10 | 1 (Ref.) | 1 (Ref.) | 1 (Ref.) |
| Individuals with bipolar disorder | 11329 | 776 | 82054 | 9.46 | **1.567 (1.418,1.731)** | **1.535 (1.386,1.701)** | **1.493 (1.345,1.656)** |
| *P* value | | | | | <0.0001 | <0.0001 | <0.0001 |
| **Hospitalization for heart failure** | | | | | | | |
| Matched^*^ controls without major psychiatric disorders | 11329 | 87 | 167698 | 0.52 | 1 (Ref.) | 1 (Ref.) | 1 (Ref.) |
| Individuals with bipolar disorder | 11329 | 82 | 86186 | 0.95 | **2.512 (1.768,3.568)** | **2.439 (1.694,3.510)** | **2.320 (1.608,3.348)** |
| *P* value | | | | | <0.0001 | <0.0001 | <0.0001 |
| **Composite of all cardiometabolic disease** | | | | | | | |
| Matched^*^ controls without major psychiatric disorders | 11329 | 5769 | 124342 | 46.40 | 1 (Ref.) | 1 (Ref.) | 1 (Ref.) |
| Individuals with bipolar disorder | 11329 | 5063 | 59493 | 85.10 | **1.940 (1.864,2.019)** | **1.880 (1.805,1.959)** | **1.858 (1.782,1.936)** |
| *P* value | | | | | <0.0001 | <0.0001 | <0.0001 |

Model 1: Unadjusted.

Model 2: Adjusted for CCI.

Model 3: Adjusted for CCI, household income, and disability.

^*^Exact 1:1 matching based on age and sex was performed.

CCI, Charlson Comorbidity Index.

**Supplementary Table 3.** Hazard ratios and 95% confidence intervals for cardiometabolic diseases and all-cause mortality in individuals with bipolar disorder treated or never treated with mood stabilizers compared with controls without major psychiatric disorders

| **Subpopulations** | **Subjects (n)** | **Events (n)** | **Follow-up duration (person-years)** | **Incidence rate (per 1,000 person-years)** | **Hazard ratio (95% confidence interval)** | | |
| --- | --- | --- | --- | --- | --- | --- | --- |
|  |  |  |  |  | **Model 1** | **Model 2** | **Model 3** |
| **Ischemic stroke** | | | | | | | |
| Matched controls without major psychiatric disorders | 11329 | 92 | 167546 | 0.55 | 1 (Ref.) | 1 (Ref.) | 1 (Ref.) |
| Individuals with bipolar disorder never treated with mood stabilizer | 3074 | 32 | 23604 | 1.36 | **1.895 (1.244,2.888)** | **1.836 (1.198,2.812)** | **1.822 (1.186,2.797)** |
| Individuals with bipolar disorder ever treated with mood stabilizer | 8255 | 49 | 62455 | 0.78 | **1.733 (1.207,2.489)** | **1.693 (1.175,2.439)** | **1.672 (1.159,2.414)** |
| *P* value | | | | | 0.0013 | 0.0029 | 0.0039 |
| **Ischemic heart disease** | | | | | | | |
| Matched controls without major psychiatric disorders | 11329 | 981 | 160754 | 6.10 | 1 (Ref.) | 1 (Ref.) | 1 (Ref.) |
| Individuals with bipolar disorder never treated with mood stabilizer | 3074 | 252 | 22314 | 11.29 | **1.477 (1.280,1.705)** | **1.455 (1.259,1.683)** | **1.442 (1.247,1.668)** |
| Individuals with bipolar disorder ever treated with mood stabilizer | 8255 | 524 | 59740 | 8.77 | **1.504 (1.347,1.680)** | **1.486 (1.329,1.662)** | **1.476 (1.319,1.652)** |
| *P* value | | | | | <0.0001 | <0.0001 | <0.0001 |
| **Hospitalization for heart failure** | | | | | | | |
| Matched controls without major psychiatric disorders | 11329 | 87 | 167698 | 0.52 | 1 (Ref.) | 1 (Ref.) | 1 (Ref.) |
| Individuals with bipolar disorder never treated with mood stabilizer | 3074 | 29 | 23664 | 1.23 | **1.815 (1.162,2.833)** | **1.783 (1.136,2.799)** | **1.772 (1.127,2.786)** |
| Individuals with bipolar disorder ever treated with mood stabilizer | 8255 | 53 | 62522 | 0.85 | **2.342 (1.626,3.374)** | **2.315 (1.602,3.345)** | **2.291 (1.583,3.314)** |
| *P* value | | | | | <0.0001 | <0.0001 | <0.0001 |
| **Composite of all cardiometabolic disease** | | | | | | | |
| Matched controls without major psychiatric disorders | 11329 | 5769 | 124342 | 46.40 | 1 (Ref.) | 1 (Ref.) | 1 (Ref.) |
| Individuals with bipolar disorder never treated with mood stabilizer | 3074 | 1463 | 15464 | 94.61 | **1.962 (1.849,2.083)** | **1.890 (1.779,2.008)** | **1.884 (1.773,2.002)** |
| Individuals with bipolar disorder ever treated with mood stabilizer | 8255 | 3600 | 44029 | 81.76 | **1.950 (1.866,2.037)** | **1.891 (1.808,1.977)** | **1.884 (1.802,1.971)** |
| *P* value | | | | | <0.0001 | <0.0001 | <0.0001 |
| **All-cause mortality during follow-up** | | | | | | | |
| Matched^*^ controls without major psychiatric disorders | 11329 | 385 | 167968 | 2.29 | 1 (Ref.) | 1 (Ref.) | 1 (Ref.) |
| Individuals with bipolar disorder never treated with mood stabilizer | 3074 | 164 | 23737 | 6.91 | **1.979 (1.637,2.394)** | **1.873 (1.544,2.272)** | **1.820 (1.498,2.211)** |
| Individuals with bipolar disorder ever treated with mood stabilizer | 8255 | 308 | 62644 | 4.92 | **2.213 (1.894,2.586)** | **2.128 (1.818,2.491)** | **2.065 (1.762,2.421)** |
| *P* value | | | | | <0.0001 | <0.0001 | <0.0001 |

Model 1: Adjusted for age and sex.

Model 2: Adjusted for age, sex, and CCI.

Model 3: Adjusted for age, sex, CCI, household income, and disability.

CCI, Charlson Comorbidity Index.

**Supplementary Table 4.** Hazard Ratios and 95% Confidence Intervals for Cardiometabolic Diseases and All-Cause Mortality in Subpopulations of Bipolar Disorder Categorized by Initial Psychiatric Diagnosis Compared With Controls Without Major Psychiatric Disorders

| **Subpopulations** | **Subjects (n)** | **Events (n)** | **Follow-up duration (person-years)** | **Incidence rate (per 1,000 person-years)** | **Hazard ratio (95% confidence interval)** | | |
| --- | --- | --- | --- | --- | --- | --- | --- |
|  |  |  |  |  | **Model 1** | **Model 2** | **Model 3** |
| **Ischemic stroke** | | | | | | | |
| Matched controls without major psychiatric disorders | 11329 | 92 | 167546 | 0.55 | 1 (Ref.) | 1 (Ref.) | 1 (Ref.) |
| Individuals with bipolar disorder initially diagnosed as depression | 4612 | 38 | 38932 | 0.98 | **1.970 (1.333,2.913)** | **1.910 (1.285,2.839)** | **1.880 (1.262,2.800)** |
| Individuals with bipolar disorder diagnosed as bipolar disorder from the onset | 6717 | 43 | 47127 | 0.91 | **1.656 (1.134,2.420)** | **1.620 (1.105,2.373)** | **1.609 (1.096,2.362)** |
| *p*-value | | | | | 0.001 | 0.0022 | 0.0031 |
| **Ischemic heart disease** | | | | | | | |
| Matched controls without major psychiatric disorders | 11329 | 981 | 160754 | 6.10 | 1 (Ref.) | 1 (Ref.) | 1 (Ref.) |
| Individuals with bipolar disorder initially diagnosed as depression | 4612 | 431 | 36540 | 11.80 | **1.905 (1.694,2.143)** | **1.881 (1.670,2.119)** | **1.867 (1.657,2.104)** |
| Individuals with bipolar disorder diagnosed as bipolar disorder from the onset | 6717 | 345 | 45514 | 7.58 | **1.176 (1.036,1.335)** | **1.161 (1.021,1.320)** | **1.151 (1.011,1.309)** |
| *p*-value | | | | | <0.0001 | <0.0001 | <0.0001 |
| **Hospitalization for heart failure** | | | | | | | |
| Matched controls without major psychiatric disorders | 11329 | 87 | 167698 | 0.52 | 1 (Ref.) | 1 (Ref.) | 1 (Ref.) |
| Individuals with bipolar disorder initially diagnosed as depression | 4612 | 35 | 39012 | 0.90 | **2.047 (1.359,3.085)** | **2.023 (1.339,3.059)** | **2.016 (1.332,3.051)** |
| Individuals with bipolar disorder diagnosed as bipolar disorder from the onset | 6717 | 47 | 47174 | 1.00 | **2.197 (1.500,3.217)** | **2.169 (1.475,3.190)** | **2.141 (1.454,3.153)** |
| *p*-value | | | | | <0.0001 | <0.0001 | <0.0001 |
| **Composite of all cardiometabolic disease** | | | | | | | |
| Matched controls without major psychiatric disorders | 11329 | 5769 | 124342 | 46.40 | 1 (Ref.) | 1 (Ref.) | 1 (Ref.) |
| Individuals with bipolar disorder initially diagnosed as depression | 4612 | 2479 | 24841 | 99.80 | **2.313 (2.202,2.430)** | **2.236 (2.127,2.351)** | **2.230 (2.121,2.345)** |
| Individuals with bipolar disorder diagnosed as bipolar disorder from the onset | 6717 | 2584 | 34653 | 74.57 | **1.700 (1.619,1.785)** | **1.648 (1.569,1.731)** | **1.640 (1.561,1.724)** |
| *p*-value | | | | | <0.0001 | <0.0001 | <0.0001 |
| **All-cause mortality during follow-up** | | | | | | | |
| Matched^*^ controls without major psychiatric disorders | 11329 | 385 | 167968 | 2.29 | 1 (Ref.) | 1 (Ref.) | 1 (Ref.) |
| Individuals with bipolar disorder initially diagnosed as depression | 4612 | 185 | 39111 | 4.73 | **1.988 (1.660,2.380)** | **1.894 (1.578,2.273)** | **1.836 (1.529,2.206)** |
| Individuals with bipolar disorder diagnosed as bipolar disorder from the onset | 6717 | 287 | 47270 | 6.07 | **2.232 (1.903,2.619)** | **2.142 (1.822,2.518)** | **2.083 (1.770,2.452)** |
| *p*-value | | | | | <0.0001 | <0.0001 | <0.0001 |

Model 1: Adjusted for age and sex.

Model 2: Adjusted for age, sex, and CCI.

Model 3: Adjusted for age, sex, CCI, household income, and disability.

CCI, Charlson Comorbidity Index.
